# Migration of Nutrient Substances and Characteristic Changes of Chicken White Soup Emulsion from Chicken Skeleton during Cooking

**DOI:** 10.3390/foods13030410

**Published:** 2024-01-26

**Authors:** Haining Guan, Yanli Tian, Chunmei Feng, Siqi Leng, Shifa Zhao, Dengyong Liu, Xiaoqin Diao

**Affiliations:** Meat Innovation Center of Liaoning Province, College of Food Science and Technology, Bohai University, Jinzhou 121013, China; hai.ning2001@163.com (H.G.); tianyanli9696@163.com (Y.T.); hrbleng@163.com (S.L.); diaoxiaoqing172@163.com (X.D.)

**Keywords:** white chicken soup, migration, nutrient substances, interfacial behavior, stability

## Abstract

The protein and fat in chicken skeleton can be emulsified in a boiling state to form milky white chicken soup. White chicken soup has a delicious taste, good nutritional value, a beautiful color, and volatile flavor compounds. However, cooking time significantly impacts the quality of white chicken soup. Herein, we investigated the influence of cooking time (30, 60, 90, 120, 150, 180, and 210 min) on the migration of nutrient substances and characteristics changes in white chicken soup from chicken skeletons. The results showed that nutrients such as total lipids, water-soluble protein, total sugars, solid matter, and oligopeptides in the chicken skeletons’ tissue continuously migrated into the soup during the cooking process. The total nutrient content in the chicken soup was highest after cooking for 180 min. Simultaneously, the white chicken soup obtained after cooking for 180 min had low interfacial tension and high whiteness, viscosity, and storage stability. The high stability index was associated with increased ζ potential and decreased particle size. The contact angle analysis results also indicated that the stability of the white chicken soup was improved when the cooking time reached 180 min. This research provides basic information for the production of high-quality white chicken soup.

## 1. Introduction

Making soup is a traditional cooking method that can dissolve the nutrients and flavor substances from the food into the soup [1]. Bone soup is widely popular due to its rich nutrients and unique flavor [2]. Zhu et al. (2021) found that the contents of water-soluble proteins, fatty acids, total sugars, and solid matters in the thermoultrasonic treatment of Greenland halibut bone soup were higher, and the micro-nano particles were smaller and more uniform, giving the bone soup better a emulsification effect and stability [3]. Chicken bones are the main by-product of chicken processing and consumption, accounting for about 15% of the weight of the chicken carcass. They contain nutrients such as collagens, calcium, fatty acids, and amino acids, as well as rich flavor compounds. However, only some of them are used as seasoning and raw materials for animal feed, while most of them are still wasted or discarded, leading to environmental pollution and reducing the economic value of the poultry industry [4]. Thus, to achieve higher productivity, the use of meat by-products is necessary [5]. Therefore, the high-value utilization of by-products has become an urgent need in the industry. Chicken bones, one of the main by-products, are excellent raw material for making soup.

The nutrients in the raw materials migrate into the soup during the stewing process and form new components. These new compounds gather together to promote the particles of the liquid gel system to form micro-nanoparticles. The binding abilities among lipids, proteins, and polysaccharides are enhanced, forming more stable colloidal micro-nanoparticles during the soup’s boiling process [6]. Gao et al. (2021) indicated that during the boiling of freshwater clam soup, a substantial number of nanoparticles, ranging in size from 40 nm to 149 nm, were formed [7]. Similarly, Ke et al. (2017) noted that porcine bone soups contain rich nanoparticles, with billions of micro-nanoparticles per milliliter of soup [8].

Current research on chicken soup has mainly focused on the preparation of clear soup, emphasizing the cooking technologies, processing skills, nutrition, and safety. However, there are limited reports on using chicken skeletons to prepare white chicken soup. Boiling chicken skeletons for an extended period allows proteins and fats to emulsify, resulting in a milky white chicken soup emulsion. However, cooking temperature also played a crucial role in the nutritional value and flavor of chicken soup [9], and the influence of temperature will be further explored in the future. This study aimed to analyze the migration of nutrients and changes in characteristics of white chicken soup during the cooking process. This analysis is expected to encourage more consumption of chicken skeletons and provide fundamental information for producing high-quality white chicken soup.

## 2. Materials and Methods

### 2.1. Materials

Fresh chicken skeletons (weight: 200 ± 15 g) without the head, neck, wings, legs, feet, breast meat, and visible fats were obtained from a farmer’s market (Jinzhou, Liaoning, China). Pure chicken bone accounted for approximately 30% of the whole chicken skeleton. All reagents used were of analytical grade and acquired from Aladdin Chemical Reagent Co., Ltd. (Shanghai, China).

### 2.2. Preparation of White Chicken Soup

Chicken skeletons stored at −20 °C were thawed overnight at 4 °C prior to use. After removing visible fat and connective tissues, the chicken skeleton was cut into small portions, then washed and drained. Then the chicken skeletons were boiled in boiling water for 5 min to eliminate the blood and gamy odor. Chopped chicken skeletons were mixed with purified water at a mass ratio of 1:2, and pre-cooked for 30 min on an induction cooker at 1300 W power (100 ± 0.26 °C), then kept stewing at 800 W (98 ± 0.47 °C) for 210 min. We took samples every 30 min from the start of stewing.

### 2.3. Migration of Nutrient Substances

#### 2.3.1. Water-Soluble Proteins

The content of water-soluble protein in the soup was determined by the biuret colorimetric method [10]. Bovine serum albumin was used as a standard sample.

#### 2.3.2. Total Sugars

Total sugars were determined using the anthrone–sulfuric acid colorimetry method [11]. Absorbance was measured at 620 nm with anhydrous glucose used as the standard. The results were expressed in mg of anhydrous glucose per mL.

#### 2.3.3. Solids

Solids were determined using the drying method. The total weight of a 20 mL sample and the weighing bottle was recorded, then dried in the oven to a constant weight. The weight of the solids was obtained by subtracting the weight of the weighing bottle from the total weight. The results were expressed in g/100 mL.

#### 2.3.4. Oligopeptides

Oligopeptides were extracted from soups according to the method of Fan et al. (2019) with minor modifications [12]. The soup (2 mL) and 2% TCA (2 mL) were mixed and centrifuged at 4000× *g* for 10 min. The supernatant (1.5 mL) was blended with 6% NaOH (1.5 mL) and a microscale biuret reagent (0.15 mL), kept at 25 °C for 15 min, and then the absorbance of the mixture was measured at 330 nm. A bovine serum albumin standard was used, and the oligopeptide content was expressed in mg/100 mL.

#### 2.3.5. Total Lipids and Composition of the Fatty Acids (FA) 

Total lipids were extracted using the procedure of Lin et al. (2020) [13]. In total, 20 mL of white chicken broth and 400 mL of chloroform: methanol (2:1, *v*/*v*) were mixed and kept at 4 °C for 24 h. The solution was then filtered using filter paper, and a NaCl solution was added. After stratification of the filtrate, the upper methanol phase was removed using a disposable syringe. The chloroform was then evaporated at 40 °C with a rotary evaporator, and the remaining substance was weighed to obtain the total lipids.

The total lipids were converted to fatty acid methyl ester (FAME) derivatives. For this, 50 mg of the total lipids was mixed with 2 mL of benzene–petroleum ether (1:1, *v*/*v*). Then, 2 mL of 14% boron trifluoride in methanol was added, and the solution was shaken at 45 °C for 30 min. Next, 2 mL of n-hexane and a saturated NaCl solution were added. After maintaining the mixture for 5 min, the upper layer was collected using a disposable syringe. This solution was filtered through a nylon syringe filter and deposited into 2 mL EP bottles (32 × 12 mm, Beijing Labgic Technology Co., Ltd., Beijing, China) for further analysis of the composition of the fatty acids using a gas chromatograph (GC).

The GC was equipped with an Agilent SP-2560 capillary column (100 m × 0.25 × 0.2 μm) and a flame ionization detector. Nitrogen was used as the carrier gas and the flow rate was 1 mL/min. The initial column temperature was 140 °C for 2 min, which was then raised to 200 °C (6 °C/min) and held for 2 min. It was then raised to 230 °C (2 °C/min) and held for 2 min, then raised to 250 °C (4 °C/min) and held for 2 min. The injection volume was 1 μL with a split ratio of 20:1. The contents of different fatty acids were obtained by comparing the GC peak areas of the external standard to the measured fatty acids. The composition of FA was expressed as μg/mL of soup.

#### 2.3.6. Sodium Dodecyl Sulphate-Polyacrylamide Gel Electrophoresis (SDS-PAGE)

The chicken soup prepared for the SDS-PAGE analysis was centrifuged. The sample was mixed with a Laemmli buffer (1:4) and heated at 99 °C for 10 min, and then run at 120 V for 60 min, with the protein marker as a reference, followed by 80 V for another 60 min. After running, the gels were stained with Coomassie Blue R-250 [14].

### 2.4. Characteristic of White Chicken Soup

#### 2.4.1. Color Analysis

Difference in chromaticity (L*, a*, b*) among the samples was measured with a color meter (CR-400, Konica Minolta, Japan). Here, L* represents brightness, a* represents red- and greenness, and b* represents yellow- and blueness. Whiteness was calculated as follows:

$$Whiteness=100-\sqrt{{\left(100-L^{*}\right)}^{2}+{\left(a^{*}\right)}^{2}+{\left(b^{*}\right)}^{2}}$$

#### 2.4.2. Zeta Potential Analysis

The ζ potential of chicken soups was measured at room temperature (approximately 25 °C) using a zeta potential analyzer (Malvern Instruments Ltd., Worcestershire, UK).

#### 2.4.3. Particle Size

Particle size was measured using a laser particle size distribution instrument (BT-9300ST, Better Instrument Co., Ltd., Dandong, China). The measurements were repeated three times for each sample.

#### 2.4.4. Rheological Measurements

The viscosity and stress of the soup were examined using a Discovery HR-1 (TA Instruments Company, New Castle, DE, USA). The parameters were set at a duration of 120.0 s at 25 °C in linear mode, with a shear rate ranging from 0.01 to 100.0 s^−1^ and a test interval of 1000 µm. The correlation between the shear rate and the shear stress was modeled by applying the power law equation as follows

τ = Kγ^n^

where τ (Pa) stands for the shear stress, γ (1/s) stands for the shear rate, K (Pa.s) stands for the consistency coefficient, and n is the flow behavior/attribute index.

#### 2.4.5. Interfacial Tension and Measurement of the Contact Angle 

Interfacial tension (IFT) was determined using the droplet shape analysis method, conducted on the OCA25 instrument (German Data Physics Instrument Co., Ltd., Filderstadt, Germany), according to the method outlined by Yang et al. (2022) with slight modifications [15]. A drop (10 μL) of the sample was injected into an optical quartz cuvette containing purified soybean oil, then positioned vertically at the end of a capillary tip (1.19 mm in diameter) at room temperature. The measurement process lasted 180 min. Changes in the interfacial tension during the stewing time of the chicken soup were assessed through an analysis of images of the water droplets. The experiment was conducted at a constant room temperature (25 °C), with a charge-coupled device camera continuously monitoring the droplets’ shape. Workstation software (SCA 20) automatically calculated the interfacial tension using the Young Laplace equation as a basis.

Chicken soup was spread to a thickness of 2 mm. Then a 5 µL water droplet was deposited onto the soup’s surface using a high-precision injector. After a 30 s equilibration period, the droplet’s image was captured with a high-speed video camera, and the droplet’s profile was numerically solved [16].

#### 2.4.6. Physical Stability

The physical stability of chicken soup complexes was monitored using a Turbiscan Lab^®^ analyzer, in accordance with the multiple light scattering theory [17]. Chicken soup samples were placed in glass tubes instantly after preparation and were monitored for 1 h, with scans occurring every minute at 25 °C. The stability of each sample was represented by the delta backscattering (∆BS) profiles as a function of the sample’s height (mm). The curves (∆BS = BS_t_ − BS_0_) were obtained by subtracting the BS profile at t = 0 h (BS_0_) from the profile at t = 1 h (BS_t_). The Turbiscan stability index (TSI) was used to evaluate each sample’s stability, with the values obtained via Turbiscan software (version 2.2.0.82-2).

#### 2.4.7. Storage Stability

To study the samples’ cold storage shelf life (4 °C), the storage stability of various soups was measured. The measurement method was adapted from Wang et al. (2016), with some modifications [18]. A 10 mL sample was placed in an observation bottle, sealed, and stored at 4 °C for intervals of 0, 7, 14, and 21 d. The upper and lower phases separated, with an emulsion phase at the bottom and a water dispersion at the top. The initial height of the total sample before storage was denoted as *H_T_*. The height of the lower phase of the samples stored for 0, 7, 14, 21 d was measured and recorded as *H_X_*. The stability index (*SI*, %) of the sample was then calculated as follows:

$$SI(\%)=\frac{H_{X}}{H_{T}}\times 100$$

### 2.5. Sensory Evaluation

White chicken soup obtained after 180 min of cooking was tested for sensory characteristics by 10 panelists (5 males and 5 females). The samples were randomly labeled and were kept between 55 and 60 °C in a water bath during testing. The trained panelists assessed the color, aroma, taste, and overall acceptability, based on a 9-point hedonic scale (1, extremely dislike; 5, neither like nor dislike; and 9, extremely like) [19]. All panelists scored the samples independently without interfering with each other, and the average value was taken as the result.

### 2.6. Statistical Analysis

All values were obtained from independent replicates performed in triplicate and are expressed as the mean ± standard error of the mean. Statistical analysis was performed using one-way analysis of variance (ANOVA) and Duncan’s multiple range test at *p* < 0.05 with IBM SPSS Statistics 19. Images were processed and produced using Origin 2019b software (OriginLab, Newton, MA, USA).

## 3. Results and Discussion

### 3.1. Migration of Nutrient Substances

**Figure 1:**
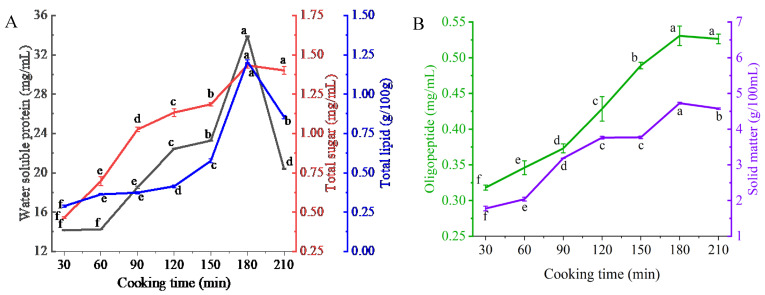
The migration of water-soluble proteins, total sugars, and total lipids (**A**), and oligopeptides and solid matter (**B**) in white chicken soups obtained after different cooking times. Error bars represent the standard errors obtained from an analysis of triplicate sample. Different letters in the same index indicate statistically significant differences (*p* < 0.05).

As shown in Figure 1A, with increased cooking time, total lipids, water-soluble proteins, and total sugars consistently migrated from the chicken skeleton to the soup. This migration significantly increased the concentration of these nutrient substances in the white chicken soup (*p* < 0.05). This increase in the constituents was consistent with the results reported for bovine bone soup [20] and salamander soup [21].

Solid matter is an important indicator of a soup’s quality as it reflects the overall nutrient content in the soup. During stewing, the solid matter released into the soup primarily derives from soluble substances, such as collagen, glycogen, minerals, and vitamins. These substances can rapidly dissolve out of chicken skeletons’ tissues, after which, the deeper tissues of the chicken skeleton release soluble substances, followed by the release of insoluble large molecules. With continuous cooking, the content of solid matter in the white chicken soup increases overall. Similar results were observed in beef soup [22]. Oligopeptides reached their highest values when the soup was boiled for 180 min (Figure 1B). This increase could be due to peptides in the meat migrating into the soup, or it could result from the degradation of proteins over longer stewing times producing oligopeptides.

However, after cooking for 180 min, these nutrient substances began to decrease, which may be attributed to further cooking causing the nutrients to degrade and interact with other components. The total lipids in white chicken soups reduced because of oxidative degradation of the lipids with continuous high-temperature treatment [23]. In the later stage of cooking, the Reducing sugars and amino acids in the soup may have undergone a Maillard reaction, resulting in a decrease in sugar substances [24]. However, there was no significant difference between the total sugar content after 180 and 210 min of cooking. It was concluded that the appropriate cooking time for white chicken soups is 180 min. In addition, the sensory evaluation results of white chicken soup obtained at this cooking time also showed that the scores for color (7.58 ± 0.38), taste (8.15 ± 0.13), and aroma (8.75 ± 0.05) were high, and the soup also received high overall acceptability scores (8.46 ± 0.08). Overall, the soup obtained after cooking for 180 min is acceptable to the consumers.

### 3.2. Composition of Fatty Acids 

**Figure 2:**
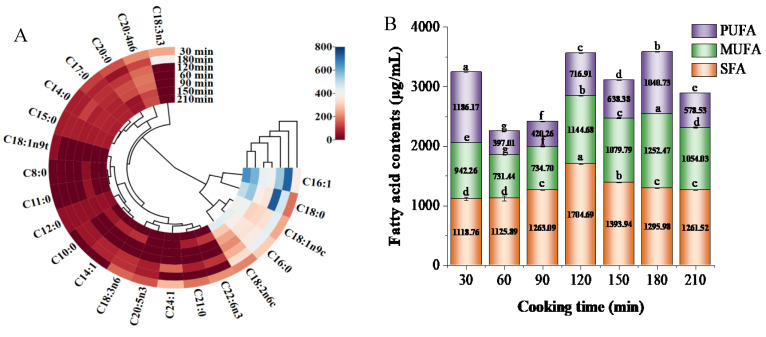
Heat map (**A**) and content analysis (**B**) of the composition of fatty acids in white chicken soups obtained after different cooking times. Error bars represent the standard errors obtained from an analysis of triplicate samples. Different letters in the same index indicate statistically significant differences (*p* < 0.05).

To study the changes in the composition of fatty acids in white chicken soup during different cooking times, a heat map was plotted (Figure 2A). The deeper the red, the lower the content, and the deeper the blue, the higher the content. In total, 22 fatty acids were detected in chicken soup, including 11 saturated fatty acids (SFAs), 5 monounsaturated fatty acids (MUFAs), and 6 polyunsaturated fatty acids (PUFAs). Among them, the main SFAs were palmitoleic acid (C16:0) and stearic acid (C18:0), and the main unsaturated fatty acids (USFAs) were oleic acid (C16:1) and oleic acid (C18:1n9c).

As shown in Figure 2B, the total SFA content in white chicken soup increased with prolonged cooking time. When the cooking time reached 120 min, the content was at its highest, but then it decreased, which may be due to degradation of the SFA into smaller molecules [25]. Meanwhile, the UFA content reached its maximum after cooking for 180 min, contributing to the formation of chicken soup flavor. Furthermore, the chicken soup cooked for 180 min offered good nutritional value in terms of fatty acids due to its higher amount of total UFAs compared with total SFAs. However, at the beginning of stewing (30 min), the chicken soup also contained high levels of PUFAs, which may be due to the migration of fatty acids from the residual meat of the chicken skeleton into the soup [26]. As the cooking time prolonged, unsaturated fatty acids were oxidized and degraded, leading to a decrease in their content [27]. When the stewing time reached 180 min, the content of unsaturated fatty acids increased again, mainly because more fat from the chicken skeleton migrated into the soup. Tufan and Koese (2014) also reported that the total PUFAs in whiting were higher than total saturated and monounsaturated FAs, indicating the high nutritional value of this species [28]. However, as the cooking time extended to 210 min, the decrease in the UFA content was attributed to the decomposition of UFAs into volatile flavor substances [29].

### 3.3. SDS-PAGE Analysis

**Figure 3:**
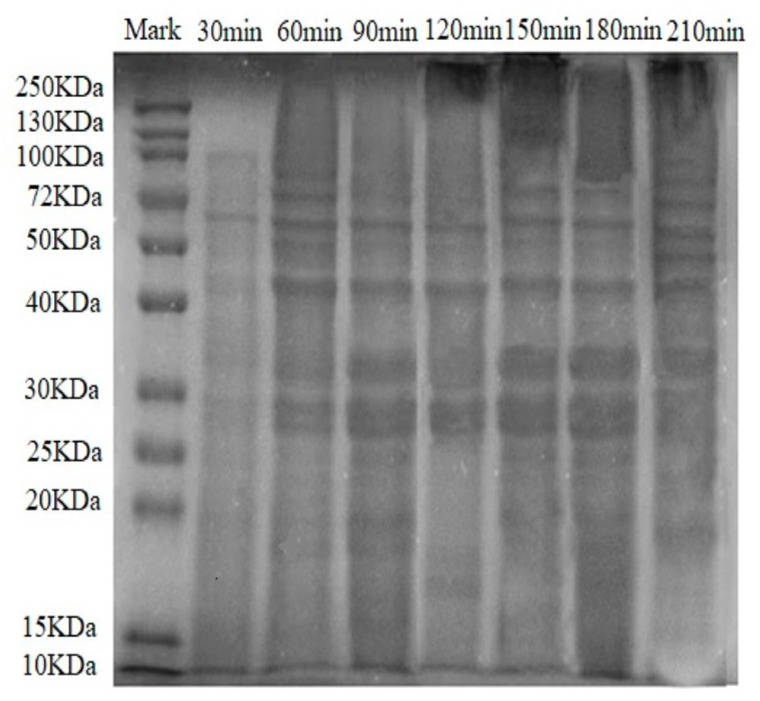
SDS-PAGE pattern of proteins in white chicken soups obtained after different cooking times. Mark: molecular weight marker.

The SDS-PAGE electrophoretogram of white chicken soup revealed different protein bands associated with various cooking times (Figure 3). The distribution of the proteins’ molecular weight in chicken soup was broad, ranging across 10–250 kDa. Figure 3 displays four major protein bands distributed around 28 kDa, 35 kDa, 44 kDa, and 60 kDa. The bands within the 25–40 kDa range matched thermostable proteins such as troponin T, troponin I, and troponin C, aligning with the findings of Qi et al. (2022) [30]. These proteins likely originated from collagen polypeptides in chicken, which were released into the soup as the connective tissue broke down during cooking [31]. The bands around 34 kDa corresponded to tropomyosin (35.2 kDa). With increased cooking times, the bands widened and deepened due to aggregation and cross-linking of the protein occurring during the cooking process. Additionally, the 40 kDa band matched actin. Sarcoplasmic protein, a water-soluble protein, comprises approximately 30% of total muscle protein in meat. It primarily consists of phosphofructokinase (82.4 kDa), phosphoglucoisomerase (60 kDa), enolase (46.4 kDa), creatine phosphokinase (44.3 kDa) and triosephosphate isomerase (28 kDa) [32]. Hence, the bands around 60 kDa matched phosphoglucoisomerase (60 kDa). After 180 min of cooking, the bands diminished and lightened in color, signifying the degradation of macromolecular proteins in the chicken soup into smaller proteins, peptides, and amino acids, or their disappearance due to oxidative decomposition during storage.

### 3.4. Characteristics of White Chicken Soup

#### 3.4.1. Color

**Figure 4:**
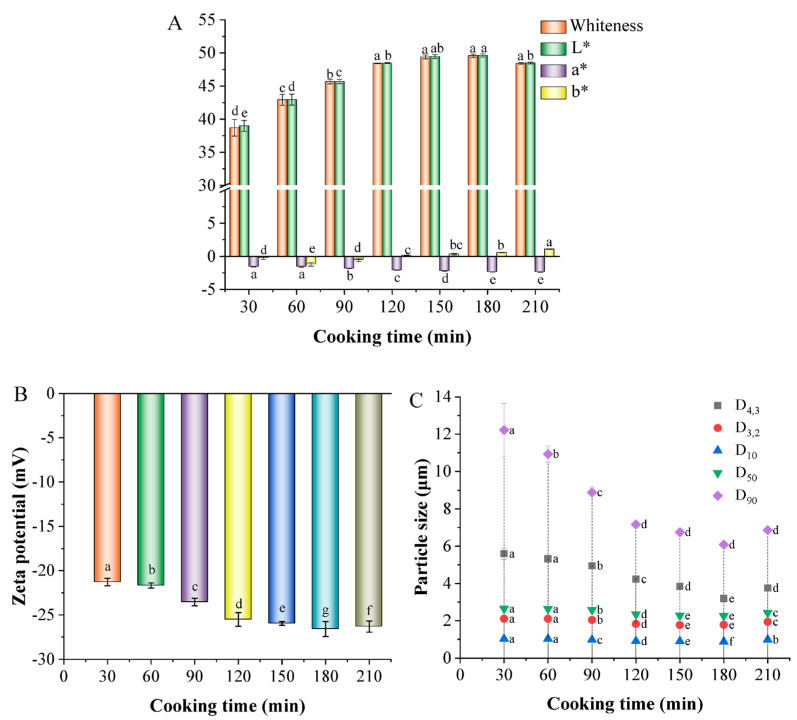
Changes in the color (**A**), zeta potential (**B**), and particle size (**C**) of chicken soups obtained after different cooking times. Error bars represent the standard errors obtained from analysis of triplicate samples. Different letters in the same index indicate statistically significant differences (*p* < 0.05).

Color is a crucial sensory indicator used to assess bone broth’s quality. As observed in Figure 4A, the L* and whiteness values of white chicken soup were higher than the a* and b* values, meaning that red and yellow were not the dominant colors in chicken soup. Typically, a large L* value indicates a tendency towards white in the sample’s color [33]. The L* and whiteness values increased with extended cooking time, peaking after 180 min. This change might be due to the long boiling time inducing the decomposition of meat tissue, resulting in a thick, white soup. Another contributing factor could be the cavitation effect caused by cooking, which breaks fat clumps in the soup into smaller globules, leading to a more evenly emulsified system [34,35]. Cooking time also promoted the migration of lipids in the soup, altering its light dispersion and enhancing its whiteness.

#### 3.4.2. Zeta Potential

The zeta potential value reveals electrostatic interactions, and the stability of the solution can be determined by measuring the magnitude of the repulsion or attraction between particles [26]. The greater the magnitude of the zeta potential (positive or negative), the higher the stability of the dispersion. A small zeta potential indicates poor stability in the dispersed system. Figure 4B displays the changes in the potential of chicken soup. As the cooking time extended, the absolute potential significantly increased. It is hypothesized that prolonged stewing leads to the formation of a stable soup system. A similar result was found by Zou, Xu, Zou, and Yang (2021), who showed that stewing techniques could increase the zeta potential of Silkie chicken soup, resulting in a stable soup system [36]. This stability occurs because larger absolute potentials strengthen the electrostatic interactions between particles [37]. In this study, when cooking time surpassed 180 min, the absolute potential decreased, signifying weakened electrostatic effects. Both electrostatic interactions and steric hindrance are crucial for maintaining chicken soup’s stability [38,39]. Hence, when the electrostatic interactions diminish, enhanced steric hindrance is necessary for maintaining the stability.

#### 3.4.3. Particle Size

Figure 4C presents the particle sizes in white chicken soup prepared for varying cooking times. As the cooking time increased, values of D10, D50, D90, D3,2 and D4,3 significantly decreased (*p* < 0.05), reaching their lowest at 180 min. This suggests that prolonged cooking facilitates the breakdown of previously formed large particles [40]. Continuous heating also gradually emulsifies the soup’s oil droplets, reducing their diameter. Qi et al. (2020) also reported that the diameter of oil droplets in emulsions were significantly larger than those of proteins [41]. Consequently, the particle size of chicken soup was primarily influenced by the size of its oil droplets. However, when the cooking time extended to 210 min, the particle sizes increased again, potentially due to the aggregation of proteins covering the oil droplets.

#### 3.4.4. Rheology

**Figure 5:**
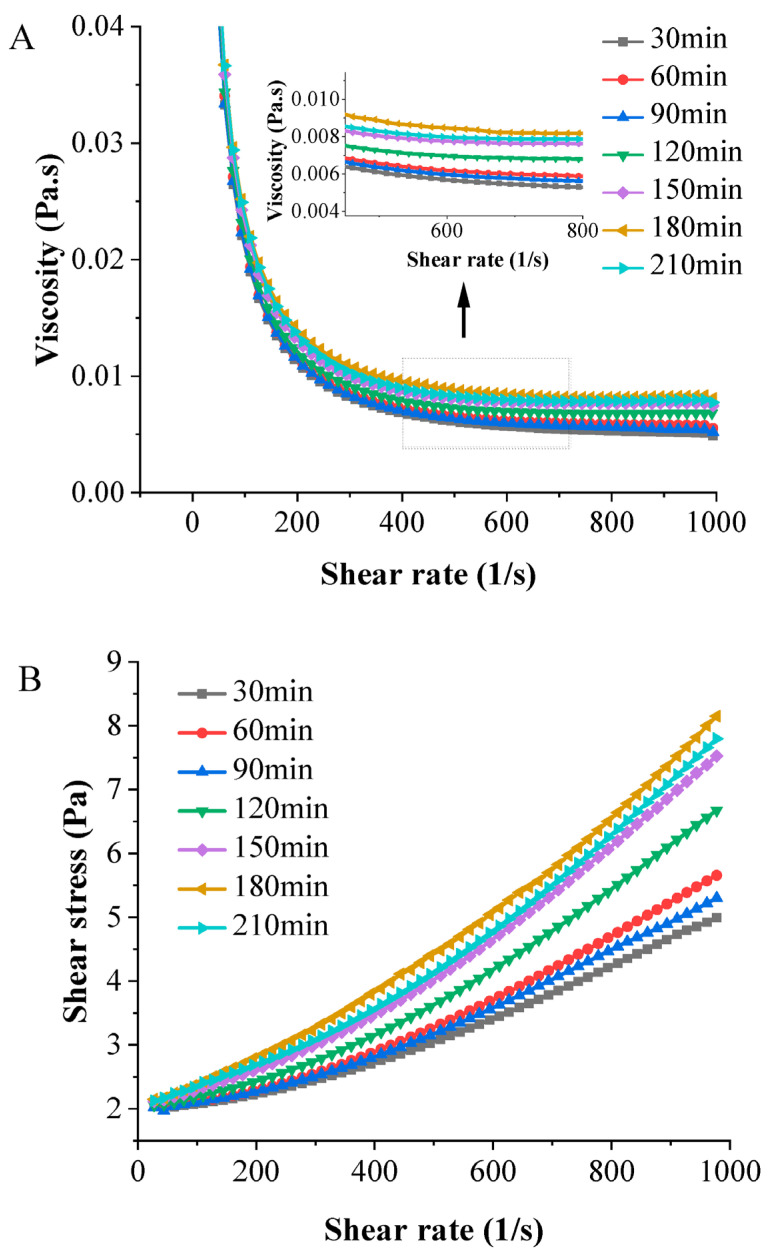
Changes in the viscosity (**A**) and shear stress (**B**) of white chicken soup obtained after different cooking times.

Rheological data are pivotal for analyzing the flow conditions during food processing [42]. Figure 5A illustrates the changes in viscosity in different chicken soups as the shear rate increased. All samples’ viscosity exhibits a linear downward trend with increasing shear rate. This decline is attributed to the disruption of oil droplet clusters in the soup, resulting in reduced shear resistance and apparent viscosity. The samples demonstrated noticeable shear-thinning behavior, primarily due to the breakdown of the interfacial layer’s network and the rearrangement of oil molecules in specific directions, decreasing the flow resistance [43]. Moreover, as the cooking time increased at a constant shear rate, the chicken soup’s viscosity rose due to the higher oil content enveloped by the proteins, affecting the soup’s network structure. Notably, a longer cooking time (210 min) reduced the chicken soup’s viscosity. This reduction in viscosity may stem from extensive protein denaturation, leading to decreased protein solubility and the formation of insoluble aggregates [44].

In addition, as shown in Figure 5B, the relationship between the shear stress and the shear rate was nonlinear, and the intercept was greater than zero. This indicates that chicken soup, under different cooking times, behaves as a non-Newtonian fluid. The curves’ intercepts were all greater than zero, highlighting the presence of yield stress in the sample. This suggests that the interactions among macromolecular substances and particle aggregations in the soup created a weak network structure [45]. White chicken soup cooked for 180 min exhibits the highest yield stress at an identical shear rate, signifying greater stability than that in the other samples.

#### 3.4.5. IFT and Contact Angle Analysis

**Figure 6:**
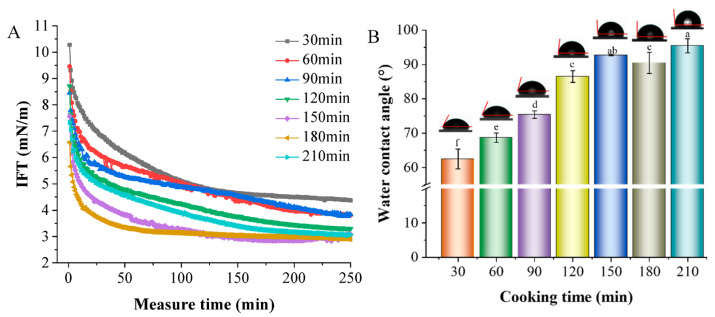
Changes in IFT (**A**) and water contact angle (**B**) of white chicken soup obtained after different cooking times. Error bars represent standard errors obtained from analysis of triplicate samples. Different letters indicate statistically significant differences (*p* < 0.05).

Interfacial tension (IFT) is a significant characteristic for studying particles’ aggregation behavior and stability in white soup at the oil/water interface, making it a crucial method for analyzing the interfacial behavior of emulsions. Reduced interfacial tension enhances the stability of the emulsion [46]. Figure 6A shows the dynamic change in IFT in white chicken soup. The IFT of different samples initially decreased, then levelled off until stable. The sample boiled for 180 min experienced the fastest and lowest drop in the IFT of the droplets, resulting in relatively stable chicken bone soup droplets. Small molecules gradually diffused to the oil–water interface, with proteins adsorbing onto it, leading to structural rearrangement. Stability in the interface layers’ interaction was achieved once the diffusion of molecules at the interface layer reached saturation. Hence, a cooking duration of 180 min is conducive to forming a stable white chicken soup system.

In oil-in-water emulsions, the particles’ interfacial wettability is vital for forming stable soup systems [47]. Figure 6B presents the contact angle (θ) of chicken soups. As the cooking time increased, the contact angle of the particles gradually rose from 62.47 to 95.47°. This indicated that the cooking time could adjust the nanoparticles’ affinity in chicken soup towards the oil phase. Previous literature has suggested [48] that particles tend to form oil-in-water emulsions when their θ values are under 90°. When the contact angle between the particles and the water phase approached 90°, the energy required for particle desorption increased, resulting in a more stable emulsion. A cooking time of 180 min brought the θ closer to 90°, enhancing the stability of chicken soup. Zhao et al. (2023) also found similar results, noting that all SiO_2_–SHP systems displayed near-neutral wetting, with θ values around 90° [49]. Thus, SiO_2_–SHP can be used as a stabilizer in the production of Pickering emulsions.

#### 3.4.6. Physical Stability

**Figure 7:**
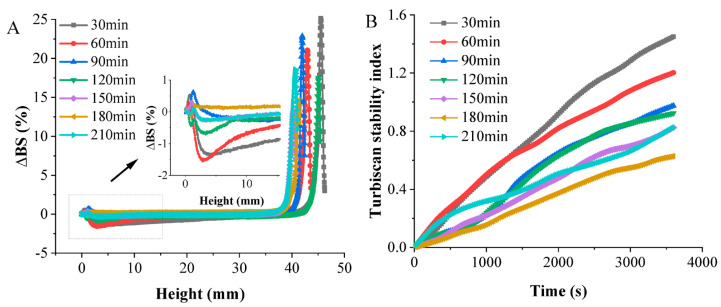
Changes in delta backscattering (**A**) at different heights (0–50 mm), and Turbiscan stability index (**B**) of white chicken soup obtained after different cooking times.

∆BS is a parameter that directly depends on the average diameter and volume fraction of droplets. Therefore, the changes in the backscattering profile of the emulsion sample are related to variations in the droplets’ size and migration processes. Typically, a positive ∆BS value signifies the migration of oil droplets, while a negative ∆BS value suggests the accumulation of droplets [50]. The change in the ∆BS value of all samples, prepared under different cooking durations, is presented in Figure 7A after the sample had been left to sit for 1 h. It was observed that the backscattered light intensity in the lower part of the chicken soup sample, following short-term cooking, decreased swiftly. This suggests that with ongoing aggregation and flocculation in the chicken soup, the droplets enlarged and ruptured. As the lower phase cleared up, the backscattered light intensity decreased, resulting in a negative ΔBS value. The ΔBS value of all soups at mid-height (30 mm) showed positive values and a minimal range of fluctuations, indicating evenly distributed particles and stability. Moreover, the ∆BS profile of the soups revealed a sharp increase in the backscattered light value (BS), indicating the accumulation of particles and a consequent positive ∆BS value. However, the ∆BS of the chicken soup prepared for 180 min of cooking was significantly lower than that of the other soups, possibly because of a thicker viscoelastic adsorption layer that stabilized the soup and hindered the process of coalescence [51]. This observation aligned with the viscosity results.

The Turbiscan stability index (TSI) is a unitless relative number enabling a comparison of the stability of different samples based on changes in the backscattering/transmission intensity over time [52]. Variations in the stability of soups cooked for different durations were tracked using the TSI value. As shown by the TSI results (Figure 7B), the TSI value for all samples increased as the measurement time progressed, indicating reduced stability. A lower TSI value signifies better stability in a system [53]. As illustrated in Figure 7B, the TSI decreased as the cooking time extended. The TSI of the chicken soup cooked for 180 min remained the lowest throughout the measurement period, implying the soup’s superior stability.

#### 3.4.7. Storage Stability

**Figure 8:**
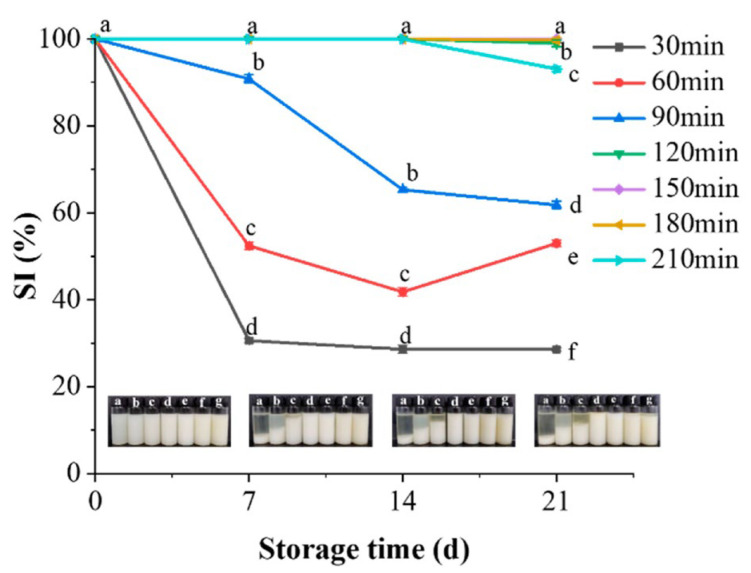
Storage stability (SI) and appearance of white chicken soup obtained after different cooking times. Error bars represent standard errors obtained from an analysis of triplicate samples. Different letters in the same sample indicate statistically significant differences (*p* < 0.05). The corresponding photographs (**a**–**g**) are of samples taken at 30, 60, 90, 120, 150, 180, and 210 min, respectively.

The influence of cooking time on the storage stability of white chicken soup is shown in Figure 8. Freshly prepared white chicken soup displayed a uniform milky white color. However, soups obtained after 30, 60, and 90 min of cooking appeared distinctly separated upon prolonged storage. The SI values of these three samples significantly decreased as the storage time extended, indicating diminished stability. In contrast, soups prepared for 150 and 180 min of cooking maintained their uniformity and stability throughout 21 d of storage. This can be attributed to the prolonged cooking times reducing particle sizes in the chicken soup, thereby enhancing the emulsification effect within the white chicken soup system. The stability of chicken soup prepared for a cooking time of 210 min commenced deterioration after 14 d in storage. This instability might result from the disruption of the previously stable system, as interactions among the water-soluble proteins, lipids, and sugars underwent multiple cycles of rupture and reconnection, leading to a weakened structural integrity and the reduced stability of the soup [54].

## 4. Conclusions

Cooking time exerts an influence on both the migration of nutrients from the chicken skeleton and the overall stability of the resulting chicken soup. In the present study, a complex colloidal emulsion system of white chicken soup was formed because of the migration of lipids, proteins, sugars, and other nutrients from chicken skeletons into the soup during 180 min of cooking. Furthermore, the white chicken soup exhibited high levels of physical and storage stability after 180 min of cooking. The study provides a theoretical basis not only for the production of high-quality white chicken soup but also for the reasonable processing of chicken skeleton by-products. Nonetheless, research into the chemical composition of the colloidal particles within the soup remains in its initial stages, and further investigation into the interactions among the components during the cooking process is warranted.

## Data Availability

Data is contained within the article.
